# Multiple Hits for the Association of Uterine Fibroids on Human Chromosome 1q43

**DOI:** 10.1371/journal.pone.0058399

**Published:** 2013-03-14

**Authors:** Brahim Aissani, Howard Wiener, Kui Zhang

**Affiliations:** 1 Department of Epidemiology, University of Alabama at Birmingham, Birmingham, Alabama, United States of America; 2 Department of Biostatistics, University of Alabama at Birmingham, Birmingham, Alabama, United States of America; Maastricht University Medical Center, The Netherlands

## Abstract

Uterine leiomyomas (or fibroids) are the most common tumors in women of reproductive age. Early studies of two familial cancer syndromes, the multiple cutaneous and uterine leiomyomatosis (MCUL1), and the hereditary leiomyomatosis and renal cell cancer (HLRCC), implicated *FH*, a gene on chromosome 1q43 encoding the tricarboxylic acid cycle fumarate hydratase enzyme. The role of this metabolic housekeeping gene in tumorigenesis is still a matter of debate and pseudo-hypoxia has been suggested as a pathological mechanism. Inactivating *FH* mutations have rarely been observed in the nonsyndromic and common form of fibroids; however, loss of heterozygosity across *FH* appeared as a significant event in the pathogenesis of a subset of these tumors. To assess the role of *FH* and the linked genes in nonsyndromic uterine fibroids, we explored a two-megabase interval spanning *FH* in the NIEHS Uterine fibroid study, a cross-sectional study of fibroids in 1152 premenopausal women. Association mapping with a dense set of single nucleotide polymorphisms revealed several peaks of association (p = 10^−2^–8.10^−5^) with the risk and/or growth of fibroids. In particular, genes encoding factors suspected (cytosolic FH) or known (EXO1 - exonuclease 1) to be involved in DNA mismatch repair emerged as candidate susceptibility genes whereas those acting in the autophagy/apoptosis (*MAP1LC3C* - microtubule-associated protein) or signal transduction (*RGS7* - Regulator of G-protein and *PLD5*– Phospoholipase D) appeared to affect tumor growth. Furthermore, body mass index, a suspected confounder altered significantly but unpredictably the association with the candidate genes in the African and European American populations, suggesting the presence of a major obesity gene in the studied region. With the high potential for occult tumors in common conditions such as fibroids, validation of our data in family-based studies is needed.

## Introduction

Uterine leiomyomas (UL) are benign neoplasms arising from the smooth muscle cells of the uterus. It is believed that these tumors develop in the majority of American women by the time they reach menopause and become symptomatic in about 25% of them [1]. Despite their benign nature, UL are responsible for significant gynecologic morbidities including excessive bleeding, pelvic pain, urinary incontinence, infertility, and pregnancy complications [2], [3]. Despite their prevalence, the epidemiology of UL has received limited research attention until recent years because of their infrequent malignant transformation. As a consequence of this morbidity, uterine fibroids are the primary indication for hysterectomy, accounting for over 600,000 hysterectomies annually in the United States [4]. Cumulative exposure to estrogen is believed to be a major etiologic factor [5] and factors that may influence the hormonal milieu, such as obesity, are also believed to be associated with the risk [6]. However, the clearly established risk factors are age (increasing risk with increasing premenopausal age), menopause (risk decreases with menopause) and African American ethnicity (higher risk compared with that of non-Hispanic Whites). There is still no adequate explanation for the higher risk among African Americans. The estimated cumulative incidence remained significantly higher for African Americans after controlling for parity and body mass index in an ultrasound screening study [7].

Compelling evidence for a genetic liability in the development of these tumors has been demonstrated in twin studies [8], [9] yet no susceptibility genes have consistently emerged from the few genome scans reported so far [10], [11]. Early studies of two dominantly transmitted Mendelian syndromes, multiple cutaneous and uterine leiomyomatosis (MCUL1; OMIM 150800), and hereditary leiomyomatosis and renal cell cancer (HLRCC; OMIM 605839) have implicated a subregion on chromosome 1q43 that contains *FH*, a gene encoding the tricarboxylic acid cycle (Krebs cycle) fumarate hydratase enzyme [12], [13].

Heterozygous germline mutations in *FH* were initially found in 25 of 42 probands in European families segregating HLRCC. These germline mutations, which included missense substitutions, protein-truncating changes and large germline deletions, were predicted to result in absent or reduced FH activity because FH functions as a homotetramer (dominant-negative effect). FH activity in some probands was reduced in comparison with activity in controls suggesting that loss of function or severe reduction in FH activity might promote tumorigenesis. These observations indicated that FH may act as a tumor suppressor that follows the classic Knudson’s ‘two-hit’ model. In line with this hypothesis, loss of heterozygosity (LOH) was observed in the skin, uterine and renal tumors from individuals with MCLU1/HLRCC. Despite these observations being supportive of a tumor suppressor function, evidence for a direct role of FH in tumorigenesis is lacking. The current paradigm invokes upregulation of hypoxia-inducible factors (HIF) and hypoxia responsive genes in tumors with reduced expression of mitochondrial FH, [14]–[17], however, the mechanism contributing to HIF activation (pseudo-hypoxic drive, increase in reactive oxygen species, defective apoptotic mechanism or anabolic drive resulting from accumulation of glycolytic intermediates) [15] is still a matter of debate.

The occurrence of inactivating mutations in *FH* and their role in predisposing women to developing the most common presentation of this condition (nonsyndromic UL; henceforth UL) have not been fully investigated. Germline mutations affecting highly conserved amino acids have rarely been observed in nonsyndromic UL [18] but loss of *FH* and linked genes on Chr.1q43 appeared as a significant event in the pathogenesis of a subset of UL [10] and of uterine leiomyosarcomas [19], the malignant counterpart of UL. Collectively, the above reports and the following observations suggested a more complex genetic architecture for UL and highlighted the need for an intensive investigation of the chromosomal region spanning *FH* and flanking gene loci: i) absence of genotype-phenotype correlations [20], [21], ii) marked genetic heterogeneity consisting of dozens of “private” *FH* mutations occurring in single individuals [20]; iii) failure to observe UL or multiple leiomyomatosis in siblings or parents of cases with FH deficiency (FHD) [13], a severe recessive neurological disorder fundamentally different from a neoplastic disorder, iv) defects in a gene with an ubiquitous expression induce tumors in a limited number of tissues [18], v) heterozygous germline mutations in the autosomally encoded mitochondrial enzyme succinate dehydrogenase (SDH) cause the inherited syndromes phaeochromocytoma and paraganglioma, implying that defects in the same energy producing pathway give rise to distinct neoplasia, and vi) candidate genes for tumorigenesis other than *FH* map to Chr.1q43. To this end, we have evaluated the association of a dense set of single nucleotide polymorphisms (SNPs) across a target 2 Mb interval with the risk and growth of UL in a cohort of premenopausal women enrolled in the National Institute of Environmental Health Sciences-Uterine Fibroid study (NIEHS-UFS). For the first time we report data suggesting a possible implication of the region linked to *FH* in the development and growth of UL in women of European and African descents.

## Methods

### Ethics Statement

The study was approved by the Human Subject’s Review boards at the NIEHS, George Washington University and University of Alabama at Birmingham. Participants gave written informed consents.

### Study Population

Detailed characteristics of the study population have been reported [22], only those relevant to the present study will be described. Briefly, a prepaid urban health plan, which is estimated to have approximately 50% African American membership and a broad socioeconomic base, was chosen for the NIEHS-UFS. A random sample of women, aged 35 to 51 years, was selected from a computerized list of members. This age range was selected because it includes late premenopausal years. Because these tumors are hormonally dependent and develop primarily in premenopausal women, the cumulative incidence estimates for the older women in this age range will approximate the lifetime risk of the development of fibroid tumors. In addition, ultrasound screening can be used for most participants in this age range because a relatively small proportion will be surgically or naturally menopausal. Demographic data were collected by self-administered questionnaire. Reproductive and gynecologic history data were collected during a telephone interview. About 92% of the study population was self-identified as African or non-Hispanic European Americans. Of the enrolled women who were premenopausal and had a diagnosis of UL (n = 1,119), 1,045 (93%) had available DNA specimens and were self-identified as African Americans (n = 574), non-Hispanic European Americans (394) and others (n = 77).

### Covariates

Information on several known or suspected risk factors has been collected in most study participants. The covariates included age, age at menarche, parity and number of pregnancies after age 25 (earlier births were not significantly related to fibroid development in the NIEHS-UFS cohort [23], [24]), body mass index (BMI) and physical activity. Education, alcohol intake and smoking have already been evaluated as potential confounders in the NIEHS-UFS and were found not to affect the physical activity or the BMI association, and, therefore, were not included in the analysis.

### Ascertainment

Fibroids status was assessed by ultrasound screening at baseline or by medical record review in 80% and 90% of the African American and European American participants. For those women who had had a pelvic ultrasound examination recently at the health plan, the radiology records from that examination were used to assess fibroid status. The remaining premenopausal participants were asked to have a pelvic ultrasound examination at the primary care site. Women for whom neither ultrasound nor medical record review could be conducted were excluded from the analyses. Both a transabdominal and a transvaginal ultrasound examination were performed. The abdominal portion evaluated fibroid change arising from the upper uterus that would not be readily seen with the transvaginal approach alone. Tumor size was classified in 3 categories of growth (small, medium and large) measured by the diameter of the tumors (1 = <2 cm, 2 = 2<4 cm, 3 = > = 4 cm).

### SNP Selection and Typing

We have explored the genetic variation over a 2 Mb-long region delimited by the *RGS7* (regulator of G-protein 7 encoding gene) and *PLD5* (phospholipase D family, member 5) genes in the HapMap reference populations (www.hapmap.org). To select haplotype tagging SNPs (tag SNPs) for typing, we followed the general approach described by Haiman et al [25]. Since NIEHS-UFS participants are essentially from African and European origins, we downloaded SNP genotype data for the target 2 Mb region from the reference HapMap populations relevant to these ethnic backgrounds; these included ASW: African ancestry in Southwest USA, CEU: Utah residents with Northern and Western European ancestry from the CEPH collection, MKK: Maasai in Kinyawa, Kenya, YRI: Yoruban in Ibadan, Nigeria. For the CEU and YRI populations, genotypes of Phase II and Phase III were downloaded. For the ASW and MKK populations, only the genotypes of Phase III were available. We then compiled a list of non-redundant SNPs from the pooled set of the 4 selected populations. Using the SNP quality assessment cutoff recommended by Illumina (either a Golden Gate validated SNP or a two-hit or HapMap validated SNP with a SNP score of at least 0.6), we further reduced the number of SNPs to a total of 3496. We then used TAGster [26] to select tag SNPs from the selected HapMap populations. Compared with the use of tag SNPs selected from each population, TAGster allows for a selection of a smaller set of final tag SNPs with no loss of power and efficiency. An r^2^ threshold of 0.80 and a minor allele frequency (MAF) of 0.05 were chosen for the SNP selection. A MAF of 5% was chosen to provide an acceptable statistical power (about 77%) to detect associations for odds greater than 2.0. A small set of multispecies conserved SNPs (MCSSNP) was selected as described [27] then filtered against the tag SNPs to exclude the redundant markers. This yielded a final set of 1853 inter-population tag SNPs (with a density of about 1 SNP per kilobase), of which 1680 had Illumina validated assays. Illumina iSelect assay was used to type the selected SNPs at the Hudson Alpha Institute for Biotechnology, Huntsville, AL.

### Quality Control

The sample quality control included DNA concentration assessment via fluorometric assay followed by integrity assessment using agarose gel electrophoresis. Samples passing quality control were then analyzed on the array using the Illumina BeadLab system to generate hybridization-ready material. Reliability in the typing data was assessed by a set of blind 82 intra- and inter-plate duplicates representative of each of the study population groups. For accurate membership assessment to the African group, we included a set of 25 unrelated YRI samples.

### Statistical Analysis

Quality control: SNP calls were checked for adherence to Hardy-Weinberg proportions (HWP) in each of the affection status and population stratum and only SNPs showing no significant deviation (p<0.01) from HWP in UL-free controls were included for analyses. Potential biases in the test statistics were evaluated by running quantile-quantile plots (Q-Q plots) within each population stratum.

### Analytical Design

Association of SNP variants with the risk and growth of UL was evaluated separately in each population stratum using dichotomous and polytomous logistic regression modeling in SAS (SAS, Cary, NC) with adjustment for the potential confounders. For the polytomous models, only the SNP tests that met the assumption for proportional odds are reported.

Prior to analysis, the membership to race groups was inferred by discriminant analysis of an extended set of SNPs (4,363 chromosome 1q and non-1q SNPs) and principal component analysis (PCA). Previous studies in NIEHS-UFS have assessed the levels of the ordinal covariates that influenced or modified the risk of UL [22], [24], [28]; we therefore modeled these covariates accordingly. Specifically, we modeled age as a continuous variable and BMI as an ordinal variable (based on categories <25, 25–29.99, 30–34.99, 35+). The other covariates were age of menarche modeled as a dichotomous variable (age < = 11 vs. other ages), exercise as a 4-level ordinal variable (lower third, middle third, next sixth, top sixth as described [22]) and parity defined as having or not having given births at age 25 and older (binary variable), the only pregnancies with protective effects in the NIEHS-UFS [24].

Multivariate-adjusted odds of UL associated with marker genotypes in PCA-defined race groups were estimated by logistic regression using the more general genotypic test (2df test). UL was modeled as a dichotomous outcome (case-control design comparing UL-free category to tumor growth categories 1,2 and 3) or as a polytomous outcome in either case-only design (comparing UL growth categories 1 vs. 2,3 and 1,2 vs. 3) or case and control design (0 vs. 1,2,3; 0,1 vs 2,3 and 0,1,2 vs 3). For the polytomous outcome, type III p-values are reported only for the SNPs that met the assumption of proportional odds. Given the dense SNP map and the expected high correlations among the SNPs, p-values were reported with no correction for multiple testing.

Because some of the covariates may function in the causal pathway(s) and thus can confound the genetic effects, sensitivity models were fitted to evaluate effect modifications by specific covariates.

We report p-values (or –Log_10_ pvalue) for type III tests of significance for the association of UL with only the marginal effects of SNP genotypes in the two major study populations (European and African Americans).

## Results

Except for a unique individual of European descent, typing was achieved in the entire study population (n = 1152), which also included subsets of 82 QC samples and 25 YRI samples. The call rate was greater than 98% in about 92% of the sampled individuals; for analysis we excluded 42 individuals (4.0%) who had call rates less than 90%. The overall concordance rate between the duplicates was 99.6%, implying that false finding due to typing errors is unlikely.

Concordant with the self-report of race, the inference of race membership by PCA yielded four distinct clusters representing populations of European, African American, other population and of African (Yoruban reference population) descents (Fig. S1; panel A). Discriminant analysis corrected population inference for several individuals (triangles) self-identified as “other populations” to either the European or the African American group (Fig. S1; panel B). Most likely these individuals are predominantly of Hispanic ethnicity. Interestingly, 16 individuals who self-identified as African Americans (AA) and 1 individual as a non-Hispanic European American (EA) clustered more closely with the Yoruban African population (YRI). This observation prompted the exclusion of this subset of 17 individuals, resulting in a total of 986 individuals (525 AA, 391 EA and 70 Other) available for analysis.

The characteristics of the participants that were available for testing the genetic association with the risk (case-control design) and tumor growth (case-only design) of UL are summarized in Table 1. Several covariates showed different distributions between the two major ethnic groups outlining the necessity to conduct separate analyses. As can be seen, there were a disproportionate number of cases and controls in the African American group, potentially affecting the study statistical power. However, compared to most UL studies, UL-free controls were clinically ascertained (at least for tumors >0.5 cm, the detection level limit by ultrasound screening) in NIEHS-UFS, suggesting that false finding due to ascertainment bias is greatly reduced. With the small size of the group defined as “other populations” likely precluding meaningful interpretations of the results, only EA and AA groups were considered for race-stratified analyses.

**Table 1:** Characteristics of the uterine fibroid study population.

	African Americans (n = 525)		European Americans (n = 391)		Other populations (n = 70)	
Characteristic	No	%	No	%	No	%
Fibroid status*						
None	132	25	196	50	28	40
Small (<2 cm)	88	17	71	18	11	16
Medium (2–<4 cm)	176	36	82	21	16	23
Large (≥4 cm)	129	25	42	11	15	21
**Age (years)**						
35–39	198	38	132	34	22	31
40–44	143	27	108	28	18	26
45–51	184	35	151	38	30	43
**Age at menarche (years)**						
<11	63	12	15	4	4	6
11	80	15	59	15	12	17
12	150	29	112	29	14	20
13	122	23	129	33	26	37
14	48	9	41	11	9	13
>14	60	12	32	8	5	7
missing	3		2			
No. Pregnancies**						
0	266	51	246	63	39	55
1	162	31	57	15	19	27
2	83	16	78	20	6	9
≥3	14	2	10	2	6	9
**Body mass index (BMI)**						
BMI = <25	132	25	226	58	28	40
25< BMI< = 30	106	20	35	9	7	10
30 = <BMI< = 35	162	31	92	23	27	39
BMI> = 35	124	24	38	10	8	11
**Physical activity**						
Low	107	27	189	36	25	36
Moderate	135	35	164	31	18	26
High	76	20	83	16	14	20
Very high	72	18	86	17	13	18

(*)diameter size;

(**)Number of full-term pregnancies after age of 25 years.

Of 1,680 assayed SNPs, 124 were excluded because the assay did not work (5 SNPs), the MAF was less than 5% (23 SNPs) or the SNP was monomorphic (96 SNPs). Examination of the proportions of p-values that were below 5% and 1% showed that the deviation from HWE (Hardy-Weinberg equilibrium) is within the nominal rate for the reference YRI population (2.2% and 0.5%, respectively). Slightly higher rates were observed in the “other populations” group; these are likely due to population structure.

**Figure 1:**
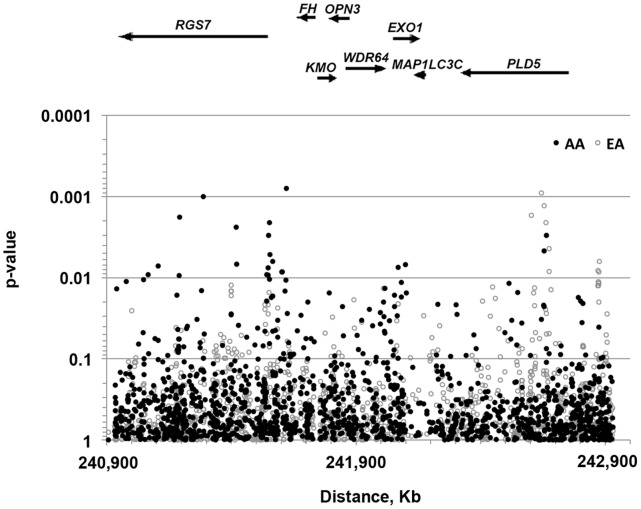
Association of chromosome 1q43 single nucleotide polymorphisms with the risk of uterine leiomyomas (unadjusted models). P-values for 2 d.f. tests from dichotomous logistic regression models adjusted for the only effect of age are reported separately for the African Americans (AA) and European Americans (EA) study groups. Because of the high correlations among the tightly linked SNPs, the p-values are not corrected for multiple testing. The placement of the genes in the gene map shown above the plot and the coordinates show below were derivedthe Human Genome assembly 19. Arrows indicate the orientation of the genes and are drawn proportionally to the size of the genes. *RGS7* (regulator of Gprotein 7); *FH* (fumarate hydratase); *KMO* (kynurenine 3monooxygenase); *OPN3* (opsin 3); *WDR64* (WD repeat domain 64); *EXO1* (exonuclease 1); *MAP1LC3C (*microtubuleassociated protein 1 light chain 3 gamma); *PLD5* (phospholipase D family, member 5).

We first evaluated the effects of the final set of SNPs (n = 1556) on the risk of UL in age-only adjusted models (together with race, age is the sole established risk factor). The association data from these models are displayed in Fig. 1 separately for the AA and EA groups. The results show at least two candidate regions of significant associations with the risk of UL in AA peaking between *RGS7* and *FH* at rs12067660 (p = 8.0×10^−4^) and in the first intron of *PLD5* in EA at rs7531009 (p = 9.0×10^−4^) and rs6429360 (p = 1.3×10^−3^). The positive association of these variants in EA or AA but not in both populations is either due to monomorphism (rs12067660 in EA) or low heterozygosities (rs7531009 and rs6429360 in AA). Additionally, two other regions reached the significance level thresholds of 0.1% and 1%; these are tagged by the intronic variant rs9787056 (p = 1.0×10^−3^ in AA) in the 5′ end of the 500 Kb-long *RGS7* gene and by rs3845563 (p = 6.9×10^−3^ in AA) and rs1776161 (p = 7.5×10^−3^ in AA) in the chromosomal interval delimited by *MAP1LC3C* (microtubule-associated protein 1 light chain 3 gamma) and *EXO1* (Exonuclease 1). Close examination of genotype frequencies for rs3845563 showed that this variant is polymorphic in both race groups (het >0.40) but slightly deviated from HWE in the AA group in both cases (p = 0.07) and controls (p = 0.08), indicating potential genotyping error or location of this variant within a copy number variation (CNV) with different population distributions.

**Figure 2:**
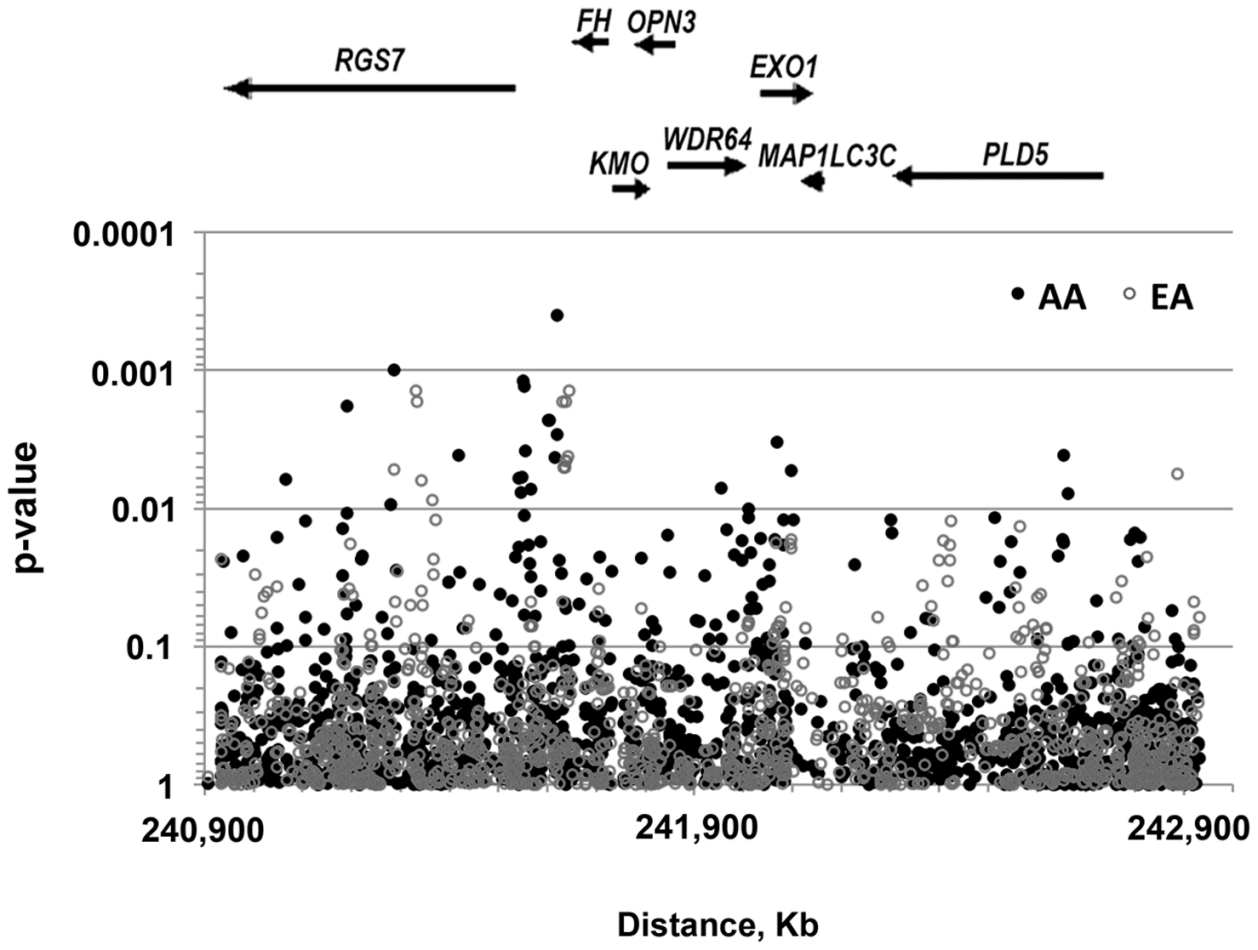
Association of chromosome 1q43 single nucleotide polymorphisms with the risk of uterine leiomyomas (**full models**)**.** P-values are from dichotomous logistic regression models adjusted for age, age at menarche, parity, physical activity and body mass index (BMI). BMI and physical activity were modeled as four-level variables and age at menarche and parity as two-level variables. Other details are as described in Fig. 1.

We then evaluated the association in models adjusting for age and for the suspected confounders (age at menarche, parity, physical activity and BMI). Adjustment for these covariates altered significantly the association pattern in EA (Fig. 2), resulting in new signals at *RGS7* and at the *RGS7-FH* intergenic interval, consistent with the pattern observed in AA, and in the drop of the association signal at the 5′ end of *PLD5* (Table S1).

Because BMI was shown to be associated with the risk of UL, albeit in a complex relationship [29] [22], we re-evaluated the genetic association in models with no adjustment for this covariate. Removing BMI from the models re-established the patterns of association observed in age-only adjusted models (Fig. S2), indicating that BMI significantly confounds the association of the tested SNPs with the risk for UL in EA but not in AA. Because the effect of BMI on the risk of UL was modified by parity in the Black Women Health Study (BWHS) [29], the most powered study to assess such multilevel effects, we tested the association in models with no adjustment for parity. Removing parity from the models led to association patterns similar to those observed in models with no adjustment for BMI (Fig. S3). Apart from the observed difference in effects of parity by race, the interpretation of these results remains difficult.

To exclude possibilities of potential biases in our data, we generated Q-Q plots to graphically assess their quality. A slight deflation in the distribution of the p-values was observed in EA but not in AA in models assessing the risk of UL (Fig. S4; panel A). It is still unclear what may have caused the deflation; the lower heterozygosity observed at a number of SNP sites in EA and the higher linkage disequilibrium among SNPs (higher correlation) in EA compared to AA are possible explanations. The deflation was not observed, however, in models assessing tumor growth (Fig. S4; panel B).

**Figure 3:**
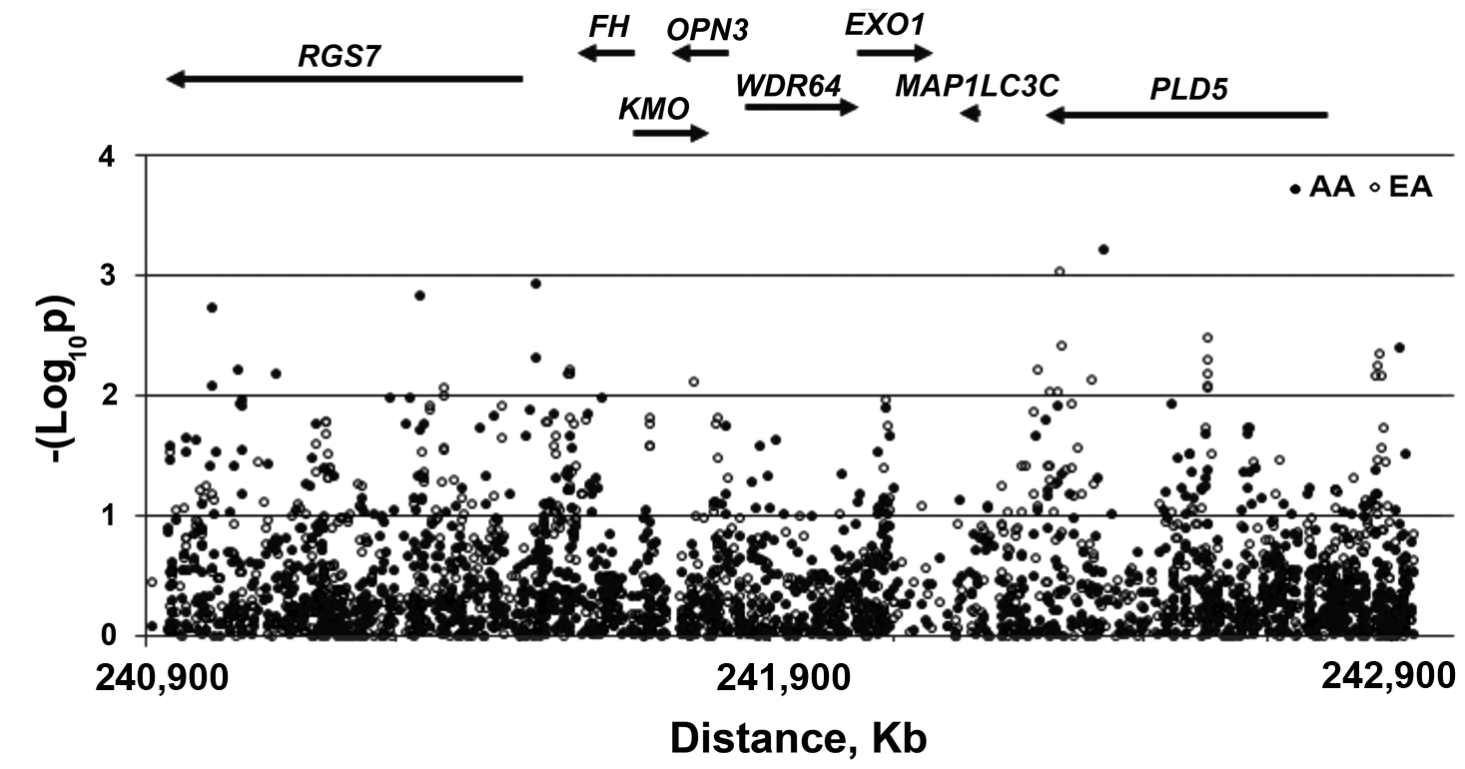
Association of chromosome 1q43 single nucleotide polymorphisms with the growth of uterine leiomyomas (affected-only design and full models). P-values are derived from polytomous logistic regression models with adjustment for the covariates as described in Fig. 2.

With our data suggesting the potential presence of more than one candidate 1q43 gene for UL, we next tested whether the studied region also affects tumor growth as measured by the diameter of the tumors. By fitting polytomous logistic regression models to our data with adjustments for the covariates in a case-only design, patterns of association similar to those obtained with the risk outcome were observed, albeit with moderately lower levels of significance (Fig. 3). By contrast, the 3′ end of *PLD5* emerged as an important candidate region for tumor growth in both populations peaking in AA at rs316912 (p = 6×10^−4^) and in EA at rs1021791 (p = 1.3×10^−3^) (Table S2). Noteworthy, rs316912 is not polymorphic in EA and has low heterozygosity in AA (het = 0.048), indicating that the small size of the samples in each category of tumor growth may have impacted the accurate estimation of the model parameters. No such low heterozygosity was observed with rs1021791, indicating that at least for EA, the *PLD5* signal may not be a false finding. The unexpected observation that the association patterns for the risk and tumor growth models are similar prompted sensitivity analyses that included the UL-free controls in the polytomous design, now comparing the 4 levels of tumor growth (0, 1, 2 and 3 for no fibroids, small, medium and large fibroids, respectively). Interestingly, higher significance levels were observed in the 4-level polytomous models (Fig. S5), particularly with a pronounced signal in AA peaking between *EXO1* and *MAP1LC3C* at rs1776161 (p = 7×10^−4^), contrasting with the vanishing association with the 3′ end of *PLD5*. It is possible that *PLD5* represents a second hit that increases the risk for tumor progression.

Having observed population-specific patterns of association, we examined whether the long-range linkage disequilibrium (LD) can explain the multiple and distinct signals of association observed in the two populations. Our results indicated a differential population pattern of LD across the extended genomic region (Fig. S6); however, there was no strong LD between very distant markers (markers that are >100 Kb apart). The data of Fig. S6 indicated that the multiple peaks of association may not be mere results of long-range disequilibria between markers and suggested that more than one putative candidate gene for UL maps in the interval separated by *RGS7* and *PLD5*. To substantiate this hypothesis, we examined the recombination rate across the tested region in the HapMap reference populations and found no extended region with low recombination rate. To the contrary, striking elevations of the recombination rate (up to 60 cM/Mb) are evident at specific hot spots along the tested region (Fig. S7).

## Discussion

The genetic basis of uterine fibroids, a condition with an estimated cumulative incidence of more than 70% for women of age 50 [30], remains largely unexplored. The present study investigated a suspected but an inadequately explored genomic region of human chromosome 1q43 for the presence of variants at risk for UL in a cohort of 1045 eligible premenopausal women. Using carefully curated high-throughput typing data and analytical models with appropriate adjustments, we show that the genomic region linked to *FH*, a gene implicated in the rare familial UL-related syndromes HLRCC and MCUL1, is also associated with the nonsyndromic and more common form of UL.

No SNP achieved statistical significance at the alpha level of 5% after correction for multiple comparisons. Although considerations such as the prior association or linkage of the studied genomic region with syndromic UL [10] [13] and nonsyndromic UL [10], the high correlation among the densely-spaced markers and the present and first replication of the association at several unlinked SNPs in the African American population argue against chance findings, independent validation of our results is needed. In contrast to the early studies of syndromic UL, our association data did not point to *FH* as the sole candidate gene and suggested alternative and/or multiple 1q43 candidate genes. These results are in line with our hypothesis that *FH* may not be the true target of inactivation in UL, at least as far as its mitochondrial isoform is concerned. It is well established that two FH isoforms, a cytosolic form and an N-terminal extended mitochondrial form, coexist in eukaryotic cells and derive from the same gene and transcript [31]. The N-terminal extended form is targeted to the mitochondrion, where the removal of the extension (mitochondrial targeting sequence or MTS) by the mitochondrial processing peptidase (MPP) generates the mature form. However the mechanism of sublocalization to the cytosol is not understood; translation at an alternate AUG site or retrograde translocation from mitochondria are possible mechanisms.

The function of the cytosolic form remains largely unknown. The enzymatic activity and level of mitochondrial and cytosolic FH in lymphoblastoid and fibroblast lines from individuals with HLRCC were reduced compared to controls [32] and no cytosolic form was detected in FH deficient fibroblast lines with different levels of remnant FH activity (<1% to about 20%) [33]. Thus it is quite possible that FH is the true target in HLRCC and MCUL1 but its tumorigenic activity is mediated by the poorly characterized and studied cytosolic form. Supportive of this hypothesis are the demonstration that dysregulation of hypoxia pathways in fumarate hydratase-deficient cells is independent of defective mitochondrial metabolism [34], and the suggested DNA repair function for cytosolic FH in yeast [35]. Interestingly, one of the most prominent signals observed in our study is precisely located in the intergenic interval separating *RGS7* and *FH* genes, a location at which the precursor *FH* variant has been mapped (GenBank: U59309.1; UniGene: Hs. 592490) (Fig. S8). It is still unclear how the cytosolic isoform is processed from the precursor mRNA; further molecular studies to characterize cytosolic FH in human cells are needed. At least in the budding yeast, cytosolic FH has been shown to be recruited to the nucleus in response to DNA damage [35] indicating that it may play a role in DNA repair.

A distinct association signal observed in one of the growth models (Fig. S5) maps close to *EXO1*, a gene encoding a class III member of the RAD2 family of endonucleases and exonucleases, generally described as a 5′3′ exonuclease. Until recently, the exact functional and biological roles of EXO1 were unclear, although this exonuclease was suspected to function in DNA replication, recombination, and repair [36]. A more recent study has demonstrated a direct role of the splice variant EXO1b in DNA damage-induced apoptosis in human and mouse fibroblasts [37]. This study has shown that EXO1 acts upstream of caspase3, DNA fragmentation and cytochrome c release and that its depletion in fibroblasts lead to a delayed DNA damage-induced apoptosis.

The relevance of these findings to our study may be substantial; indeed, since fibroids rarely develop into malignant tumors and eventually will shrink sometime prior to menopause or afterwards, delayed DNA damage-induced apoptosis would be an attractive model for the pathogenesis of UL. Several non-synonymous SNPs and non-coding SNPs in the gene promoter including rs10802996, a SNP associated with cervical cancer [38], and in the 3′ UTR sequences to downstream of *EXO1* showed significant associations with the risk of UL and the 4-level tumor growth outcome.

Two other genes of potential relevance to tumor growth, *MAP1LC3C* (microtubule associated protein 1 light chain 3 gamma) and *PLD5* (phospholipase D family, member 5) colocalize to chromosome 1q43. *MAP1LC3C* encodes an ortholog of the yeast autophagosome protein Atg8 [39], which is essential for autophagy. Both oncogenesis and tumor survival are influenced by perturbations of the molecular machinery that controls autophagy. Thus, the possibility that a defective MAPLC3 promotes profibrotic phenotypes and tumor progression cannot be excluded.

PLD5 is the most recently identified member of the PLD family. The best characterized members are PLD1 and PLD2 which occur as splice variants [40]. Several lines of evidence for a link between PLD activities and mitogenic signaling as well as suppression of apoptosis have been reported [41]. In addition to HLRCC and MCLU1, another cancer phenotype maps to the studied region (Predisposing for Prostate Cancer or PCaP; OMIM 602759). PCaP is an early-onset hereditary prostate cancer mapped to 1q42.3q43 in families from Southern Europe [42]. Further refinement of the linkage [43] placed the putative prostate locus in the interval between D1S321 (telomeric to *CHML* at position 241.82 Mb) and D1S2842 (telomeric to *PLD5* at 242.87 Mb). Only few follow-up studies were reported and replication of this finding has been difficult [44]. Further studies are needed to understand whether chromosome 1q43 encodes genetic factors commonly altered in PCaP and UL.

While the relevance of *FH*, *EXO1*, *MAP1LC3*C and *PLD5* functions to the development of cancer is plausible, that of the associated *RGS7* is intriguing and deserves comments. *RGS7* encodes a member of the regulator of G-protein signaling involved in cellular signaling and is predominantly and highly expressed in the brain. To our knowledge, no evidence for the association of RGS7 with cancer has so far been reported. Our early linkage scan for obesity loci in the Quebec Family Study has highlighted *RGS7* as a potential candidate gene for body fat in individuals of European descent [45]. We have replicated the obesity findings in the NIEHS study and our data suggested potential confounding effects due to linkage disequilibrium between genes affecting UL and obesity (Aissani, unpublished data).

**Figure 4:**
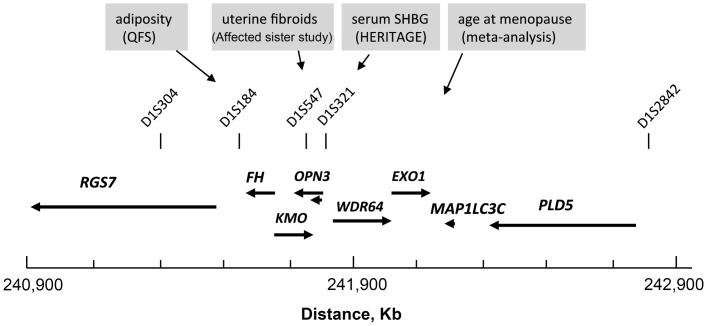
Colocalization of human traits of potential relevance to uterine fibroids on human chromosome 1q43. Genomic map of a 2 megabase-long interval spanning the fumarate hydratase (*FH*) gene and showing the position of specific microsatellite markers (vertical bars) and gene loci linked or associated with diseases or traits of relevance to hormone-dependent tumors such as uterine leiomyomas. QFS (Quebec family study); SHBG (sex hormone binding globulin); HERITAGE (HERITAGE family study). Other details are as described in Fig. 1.

The present data as well as those from studies that have implicated the genomic region studied here in the variation of plasma levels of sex-hormone binding globulin [46], age at menopause [47] and obesity [48] (Fig. 4) converge on the idea that this region may host important genes involved in energy homeostasis and tumorigenesis. A candidate gene linking energy homeostasis and cancer would be *FH* whereby a single inactivating mutation can affect distinct functions encoded by the same gene (cytosolic *FH* with DNA repair activity and mitochondrial *FH* with metabolic activity). This model is not inconsistent with the idea that other 1q43 genes may interact with *FH* to increase the risk for or growth of UL. An alternative model is that the genes clustered with the UL gene and affecting the correlated phenotypes (serum SHBG level, age at menopause and obesity) would merely confound the association by virtue of their tight LD with the causal gene variants [48]. This model is attractive as it may provide a possible genetic explanation to the inverse J-shaped pattern of association between UL and obesity.

Here, we have shown that the genomic region of chromosome 1q43 linked to *FH* encodes one or more factors potentially influencing the risk and growth of uterine fibroids. Like for the syndromic counterparts of UL, *FH* remains a strong candidate gene for nonsyndromic UL. The discovery of HLRCC and MCUL1, two allelic and rare inherited neoplastic syndromes with a high penetrance for UL, has provided an unprecedented opportunity to map the linked chromosomal region and identify candidate loci. The importance of this discovery is worth an emphasis. Indeed, it is quite possible that genome-wide scans for nonsyndromic UL such as that reported for the Japanese population [11] could have missed chromosome 1q43 susceptibility variants because of i) the complex etiology of common diseases with low penetrant alleles, ii) high recombination rate across 1q43 necessitating dense marker maps to identify causal variants and 3) the paucity of cohort studies with clinically ascertained UL-free controls.

The paucity of genetic epidemiologic studies of UL represents a major obstacle to improving our understanding of UL pathogenesis. Apart from being an underfunded research area, the design of epidemiologic studies of UL suffers essentially from ascertainment bias due to the difficulty to define UL-free controls in cross-sectional studies ([6]; for a review). The present study was conducted in the NIEHS cohort, one of the rare epidemiologic studies of UL that clinically ascertained UL-free controls; indeed, 80% and 90% of the African American and European American participants were screened by ultrasounds at baseline.

We have observed differences in the identity of the most significantly associated SNPs between the two major study populations; these results are not inconsistent with the predicted difference in the LD pattern between tag and causal SNPs or with a possible genetic heterogeneity.

Analyses are under way to evaluate the interactions among the 1q43 SNP variants associated with UL and with the correlated phenotypes that map to the studied region (obesity and age at menopause/menarche). New insights into the pathogenetic mechanism of UL may be gained through examination of long-range haplotypes across the clustered candidate 1q43 genes that may carry independent risk alleles with incremental effects. Of note, the very high recombination rate (up to 60 cM/Mb) over the studied region suggests a profound impact of positive selection on this part of the genome. Because reproduction is a trait under strong selection, the candidate 1q43 UL gene may well be a gene affecting reproduction.

## Supporting Information

Figure S1 **Principal component analysis and race membership.** Discriminant analysis was used to group self-identified participants on the basis of 4,363 single nucleotide polymorphisms and two principal components (panel A). Color indicates self-identified race as African Americans (AA), European Americans (EA), Other populations (Oth) and Yoruban reference African population (YRI). Corrected race membership was inferred by discriminant analysis using three principal components (Panel B).
(TIF)

Figure S2 **Association of chromosome 1q43 single nucleotide polymorphisms with the risk of uterine leiomyomas (reduced models, no body mass index).** P-values for 2 d.f. tests from dichotomous logistic regression models adjusted for age, age at menarche, parity and physical activity are reported separately for the African Americans (AA) and European Americans (EA) study groups. Physical activity was modeled as a four-level variable and age at menarche and parity as two-level variables. Because of the high correlations among the tightly linked SNPs, the p-values are not corrected for multiple testing. The coordinates in the gene map shown above the plot were derived from the Human Genome assembly 19. Arrows indicate the orientation of the genes and are drawn proportionally to the size of the genes. *RGS7* (regulator of G-protein 7); *FH* (fumarate hydratase); *KMO* (kynurenine 3-monooxygenase); *OPN3* (opsin 3); *WDR64* (WD repeat domain 64); *EXO1* (exonuclease 1); *MAP1LC3C (*microtubule-associated protein 1 light chain 3 gamma); *PLD5* (phospholipase D family, member 5).
(TIF)

Figure S3 **Association of chromosome 1q43 single nucleotide polymorphisms with the risk of uterine Leiomyomas (reduced models, no parity).** P-values for 2 DF test from dichotomous logistic regression models with adjustment for age, age at menarche, physical activity and body mass index (BMI) are reported separately for the African Americans (AA) and European Americans (EA) study groups. Other details are as described in Fig. S2.
(TIF)

Figure S4 **Quantile-Quantile plots for the risk and growth of uterine leiomyomas (UL).** Q–Q plots are depicted separately for each population stratum. P-values for the observed and expected distributions are from 2d.f. test of significance plotted as logarithm decimal values. The Q–Q plots show a slight deflation of the distribution for the risk of UL in European Americans (panel A) but not for tumor growth (Panel B).
(TIF)

Figure S5 **Association of chromosome 1q43 single nucleotide polymorphisms with the growth of uterine leiomyomas (full models, affected and non-affected designs).** P-values are derived from 4-level polytomous logistic regression models with adjustment for the covariates as described in Fig. S2. Other details are as described in Fig. S2.
(TIF)

Figure S6 **Long-range pattern of linkage disequilibrium across the target 1q43 genomic region.** PLINK plots of the pattern of pairwise linkage disequilibrium (LD) along the studied region shown in the centromere to telomere direction. Islands of useful LD (haploblocks) are evident but differ in magnitude and extent in the European Americans (EA) and African Americans (AA) populations, specifically in the region around *FH* (arrow) located in the genomic region telomeric to *RGS7* (gene surrounded by a box).
(TIF)

Figure S7 **Recombination rate across the target 1q43 genomic region.** Data for the recombination rate across the studied region was derived from the HapMap database. Hot spots for recombination (up 60 cM/Mb compared to the genome average of 23 cM/Mb) are present all over the shown region; they highlight the necessity to type dense set of markers for the fine mapping of causal variants.
(TIF)

Figure S8 **Genomic map of alternative splice variants of fumarate hydratase transcripts**. The map shows the location of the precursor mRNA (Hs.592490) and the shorter splice variants of fumarate hydratase along with that of single nucleotide polymorphism rs12067660 found to be in significant association with the risk of uterine fibroids (p = 4.0×10^−4^; Fig. 1).
(TIF)

Table S1 List of DNA variants associated* with the risk of uterine leiomyomas. (*) Only SNPs reaching significant levels of association (p≤0.01) in at least one of the race strata, African Americans (AA) or European Americans (EA), are reported. (ns) not significant at α = 0.05 in either EA or AA, or at α = 0.01 in both EA and AA. (nd) not determined. (no BMI) not adjusted for the effect of body mass index. (Upstream or downstream −2 Kb) SNP located within 2-kilobase distance from the transcription initiation site and the 3′ UTR, respectively. (§) Missense nucleotide substitution at codon 76 (Val76Ile). (¶) SNP rs10802996 was shown to be associated with cervical cancer [35].
(DOCX)

Table S2 List of DNA variants associated* with the growth of uterine leiomyomas. Data are from logistic models for uterine leiomyoma case-only design. (*) Only SNPs reaching significant levels of association (p≤0.01) in at least one of the race strata and meeting the assumption for proportional odds are reported. Of note, most of the SNPs that did not meet this assumption were not significant at α = 5%. (AA) African Americans; (EA) European Americans (EA). (ns) not significant at α = 5% in either EA or AA, or at α = 0.01 in both EA and AA. (nd) not determined. (no BMI) not adjusted for the effect of body mass index. (Upstream-2 Kb) SNP located within 2-kilobase distance from the transcription initiation site. (¶) synonymous nucleotide substitution at codon 23 (Cys23Cys).
(DOCX)
