# Correction: Multiple Hits for the Association of Uterine Fibroids on Human Chromosome 1q43

**DOI:** 10.1371/journal.pone.0118716

**Published:** 2015-03-18

**Authors:** The PLOS ONE Staff

There are errors in Table 1. Many values are inadvertently switched in the Body mass index (BMI) rows and the Physical activity rows. Please see the corrected Table 1 here.

**Table 1:** Characteristics of the uterine fibroid study population.

	African Americans (n = 525)		European Americans (n = 391)		Other populations (n = 70)	
Characteristic	No	%	No	%	No	%
Fibroid status*						
None	132	25	196	50	28	40
Small (<2 cm)	88	17	71	18	11	16
Medium (2–<4 cm)	176	36	82	21	16	23
Large (≥4 cm)	129	25	42	11	15	21
**Age (years)**						
35–39	198	38	132	34	22	31
40–44	143	27	108	28	18	26
45–51	184	35	151	38	30	43
**Age at menarche (years)**						
<11	63	12	15	4	4	6
11	80	15	59	15	12	17
12	150	29	112	29	14	20
13	122	23	129	33	26	37
14	48	9	41	11	9	13
>14	60	12	32	8	5	7
missing	3		2			
No. Pregnancies**						
0	266	51	246	63	39	55
1	162	31	57	15	19	27
2	83	16	78	20	6	9
≥3	14	2	10	2	6	9
**Body mass index (BMI)**						
BMI ≤ 25	132	25	226	58	28	40
25 < BMI ≤ 30	163	31	92	23	27	39
30 ≤ BMI ≤ 35	106	20	35	9	7	10
BMI ≥ 35	124	24	38	10	8	11
**Physical activity**						
Low	189	36	107	27	25	36
Moderate	164	31	135	35	18	26
High	83	16	76	20	14	20
Very high	86	17	72	18	13	18
Missing	3		1			

(*) diameter size;

(**) Number of full-term pregnancies after age of 25 years
