# Supplementary figures and images for: Multiple Hits for the Association of Uterine Fibroids on Human Chromosome 1q43
Source: PLoS One. 2013 Mar 14;8(3):e58399. doi: 10.1371/journal.pone.0058399 (PMC3604173; doi:10.1371/journal.pone.0058399)

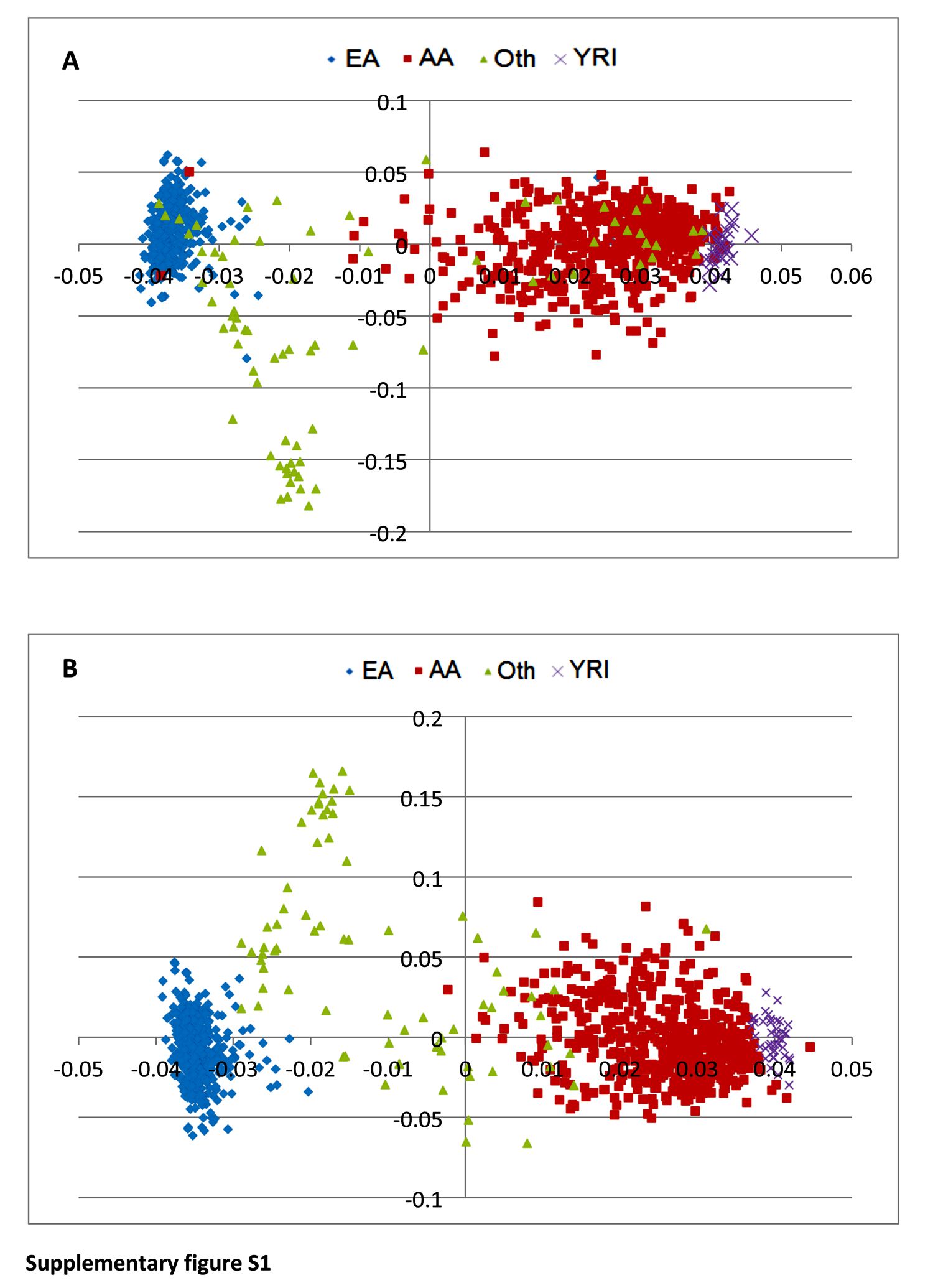

Supplement: Figure S1 — Principal component analysis and race membership. Discriminant analysis was used to group self-identified participants on the basis of 4,363 single nucleotide polymorphisms and two principal components (panel A). Color indicates self-identified race as African Americans (AA), European Americans (EA), Other populations (Oth) and Yoruban reference African population (YRI). Corrected race membership was inferred by discriminant analysis using three principal components (Panel B). (TIF) [file pone.0058399.s001.tif]

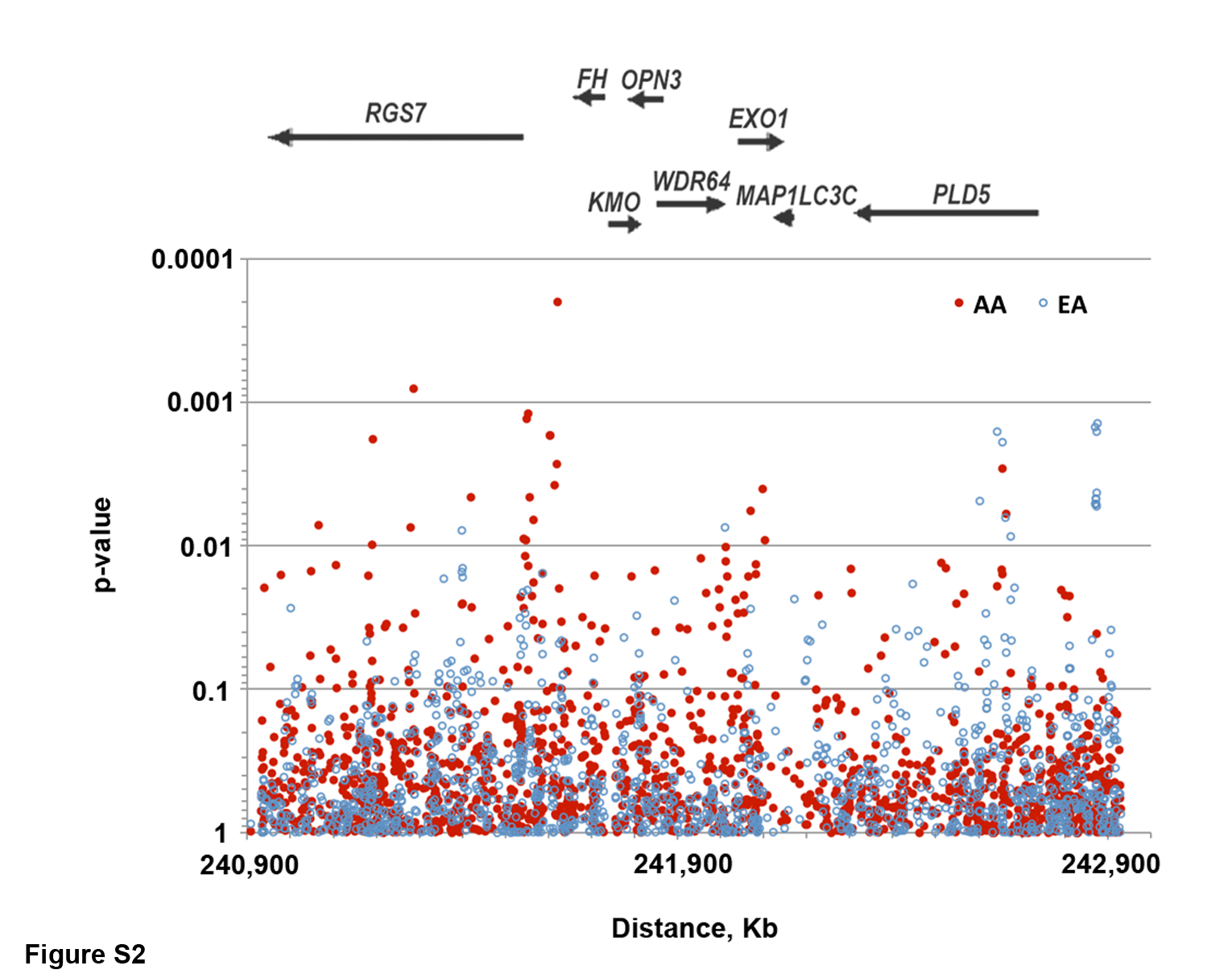

Supplement: Figure S2 — Association of chromosome 1q43 single nucleotide polymorphisms with the risk of uterine leiomyomas (reduced models, no body mass index). P-values for 2 d.f. tests from dichotomous logistic regression models adjusted for age, age at menarche, parity and physical activity are reported separately for the African Americans (AA) and European Americans (EA) study groups. Physical activity was modeled as a four-level variable and age at menarche and parity as two-level variables. Because of the high correlations among the tightly linked SNPs, the p-values are not corrected for multiple testing. The coordinates in the gene map shown above the plot were derived from the Human Genome assembly 19. Arrows indicate the orientation of the genes and are drawn proportionally to the size of the genes. RGS7 (regulator of G-protein 7); FH (fumarate hydratase); KMO (kynurenine 3-monooxygenase); OPN3 (opsin 3); WDR64 (WD repeat domain 64); EXO1 (exonuclease 1); MAP1LC3C (microtubule-associated protein 1 light chain 3 gamma); PLD5 (phospholipase D family, member 5). (TIF) [file pone.0058399.s002.tif]

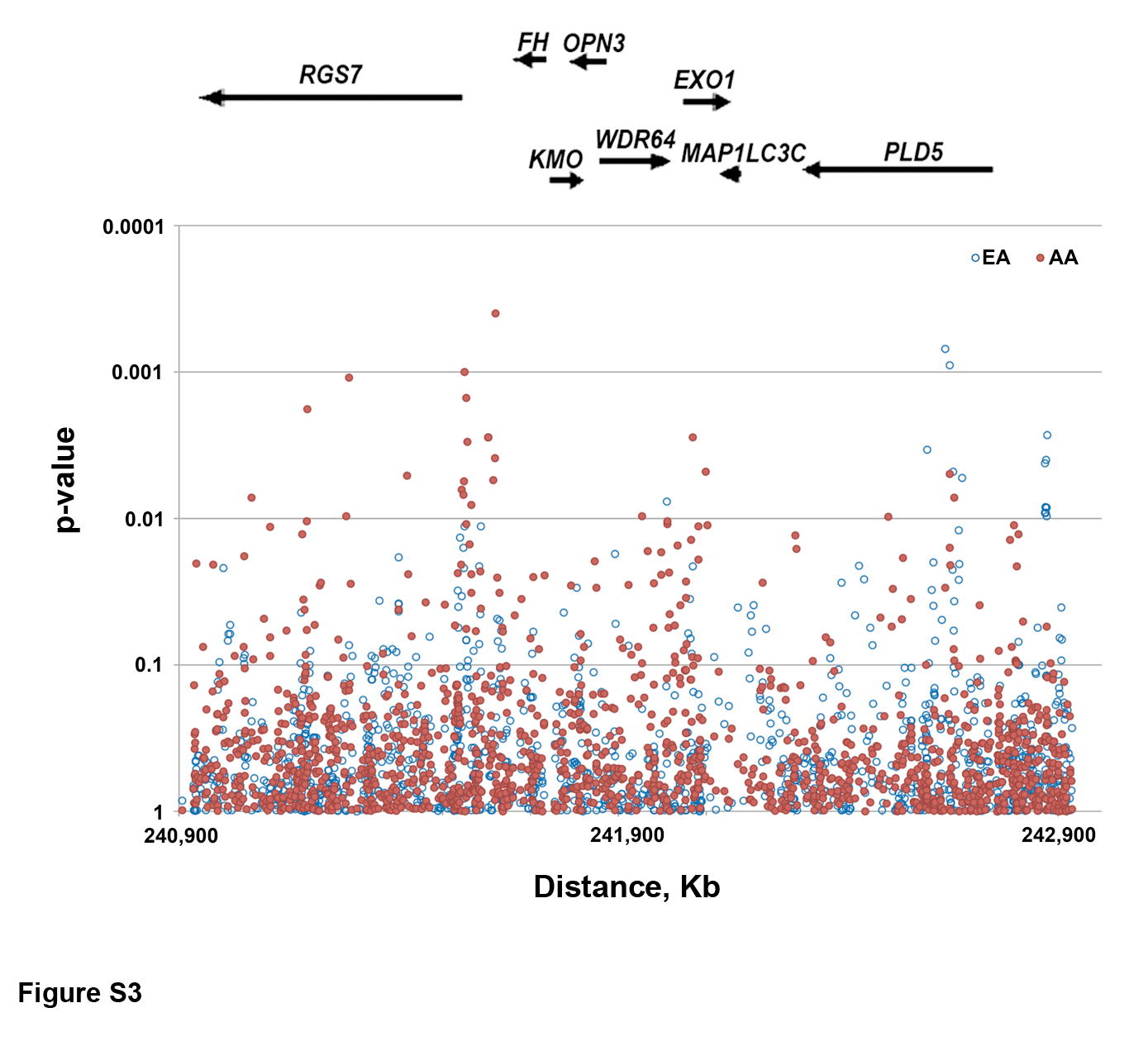

Supplement: Figure S3 — Association of chromosome 1q43 single nucleotide polymorphisms with the risk of uterine Leiomyomas (reduced models, no parity). P-values for 2 DF test from dichotomous logistic regression models with adjustment for age, age at menarche, physical activity and body mass index (BMI) are reported separately for the African Americans (AA) and European Americans (EA) study groups. Other details are as described in Fig. S2. (TIF) [file pone.0058399.s003.tif]

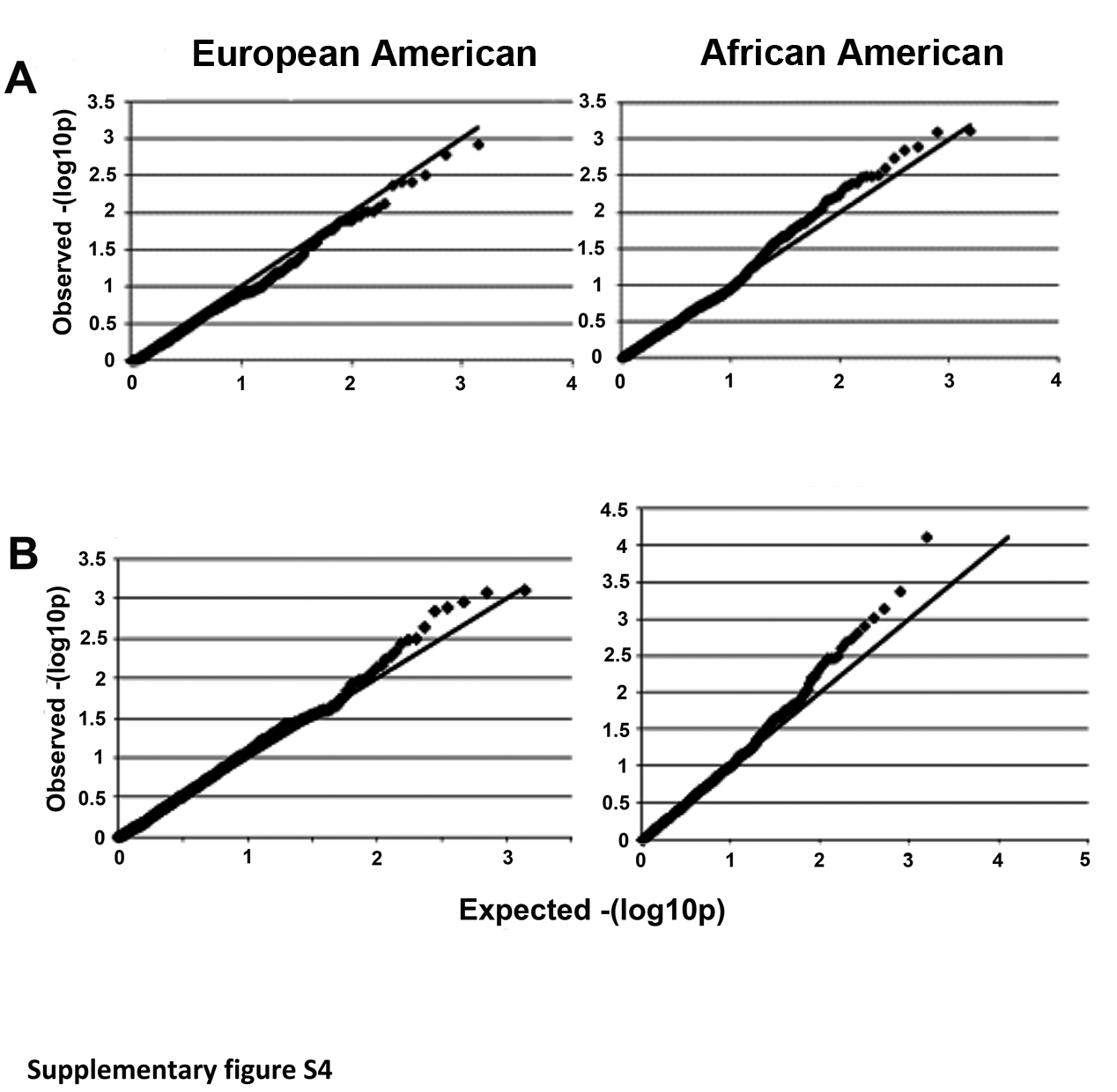

Supplement: Figure S4 — Quantile-Quantile plots for the risk and growth of uterine leiomyomas (UL). Q–Q plots are depicted separately for each population stratum. P-values for the observed and expected distributions are from 2d.f. test of significance plotted as logarithm decimal values. The Q–Q plots show a slight deflation of the distribution for the risk of UL in European Americans (panel A) but not for tumor growth (Panel B). (TIF) [file pone.0058399.s004.tif]

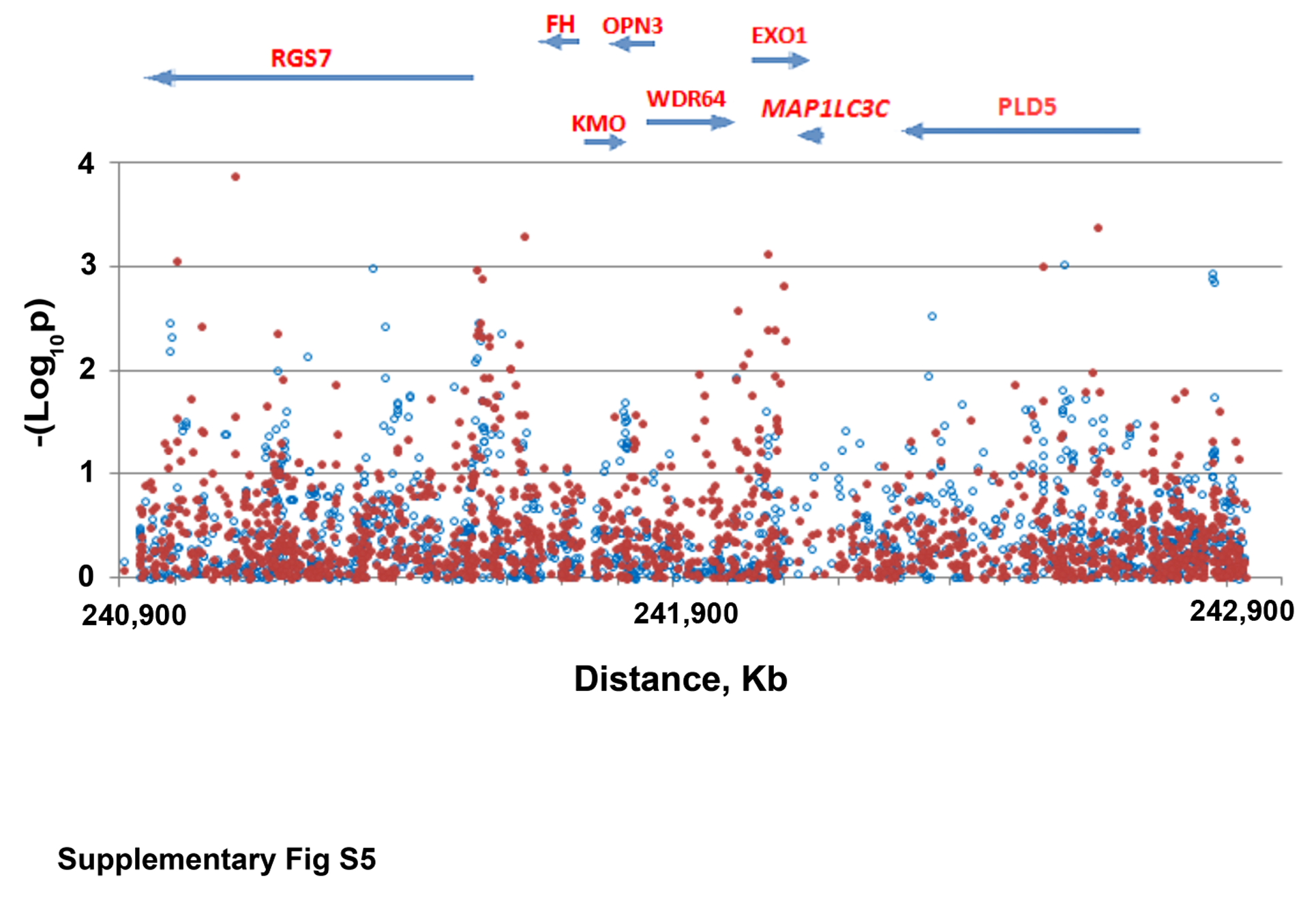

Supplement: Figure S5 — Association of chromosome 1q43 single nucleotide polymorphisms with the growth of uterine leiomyomas (full models, affected and non-affected designs). P-values are derived from 4-level polytomous logistic regression models with adjustment for the covariates as described in Fig. S2. Other details are as described in Fig. S2. (TIF) [file pone.0058399.s005.tif]

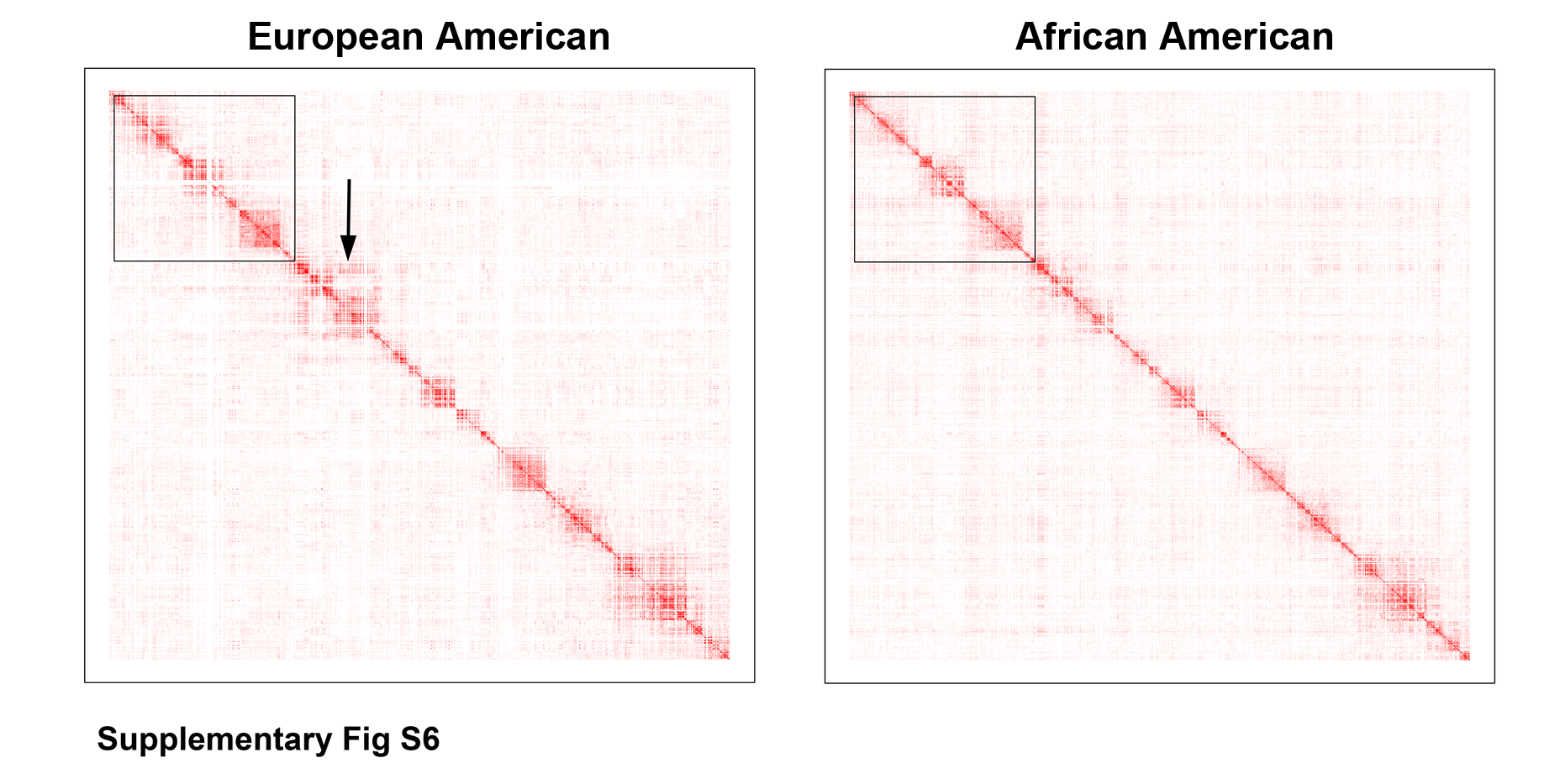

Supplement: Figure S6 — Long-range pattern of linkage disequilibrium across the target 1q43 genomic region. PLINK plots of the pattern of pairwise linkage disequilibrium (LD) along the studied region shown in the centromere to telomere direction. Islands of useful LD (haploblocks) are evident but differ in magnitude and extent in the European Americans (EA) and African Americans (AA) populations, specifically in the region around FH (arrow) located in the genomic region telomeric to RGS7 (gene surrounded by a box). (TIF) [file pone.0058399.s006.tif]

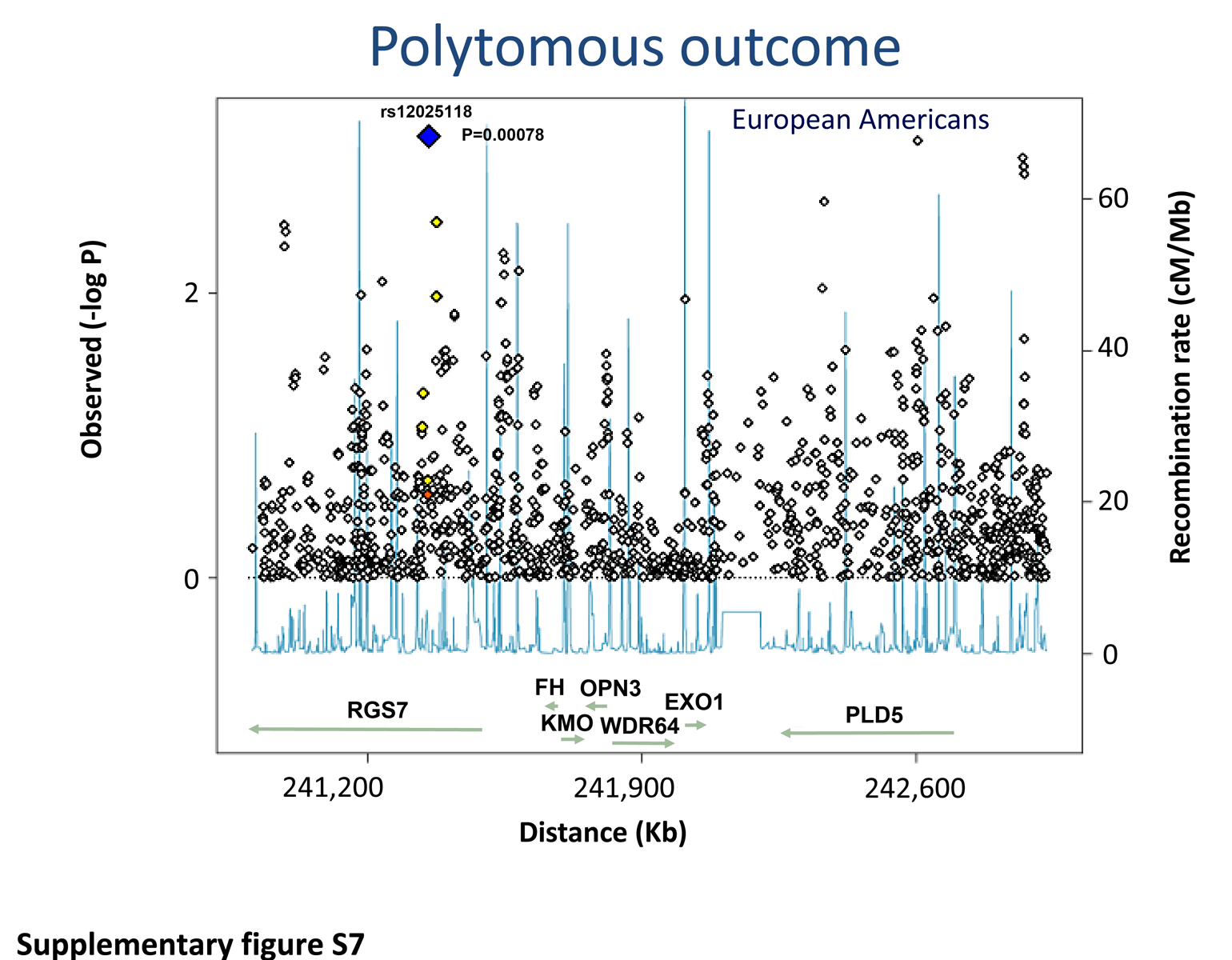

Supplement: Figure S7 — Recombination rate across the target 1q43 genomic region. Data for the recombination rate across the studied region was derived from the HapMap database. Hot spots for recombination (up 60 cM/Mb compared to the genome average of 23 cM/Mb) are present all over the shown region; they highlight the necessity to type dense set of markers for the fine mapping of causal variants. (TIF) [file pone.0058399.s007.tif]

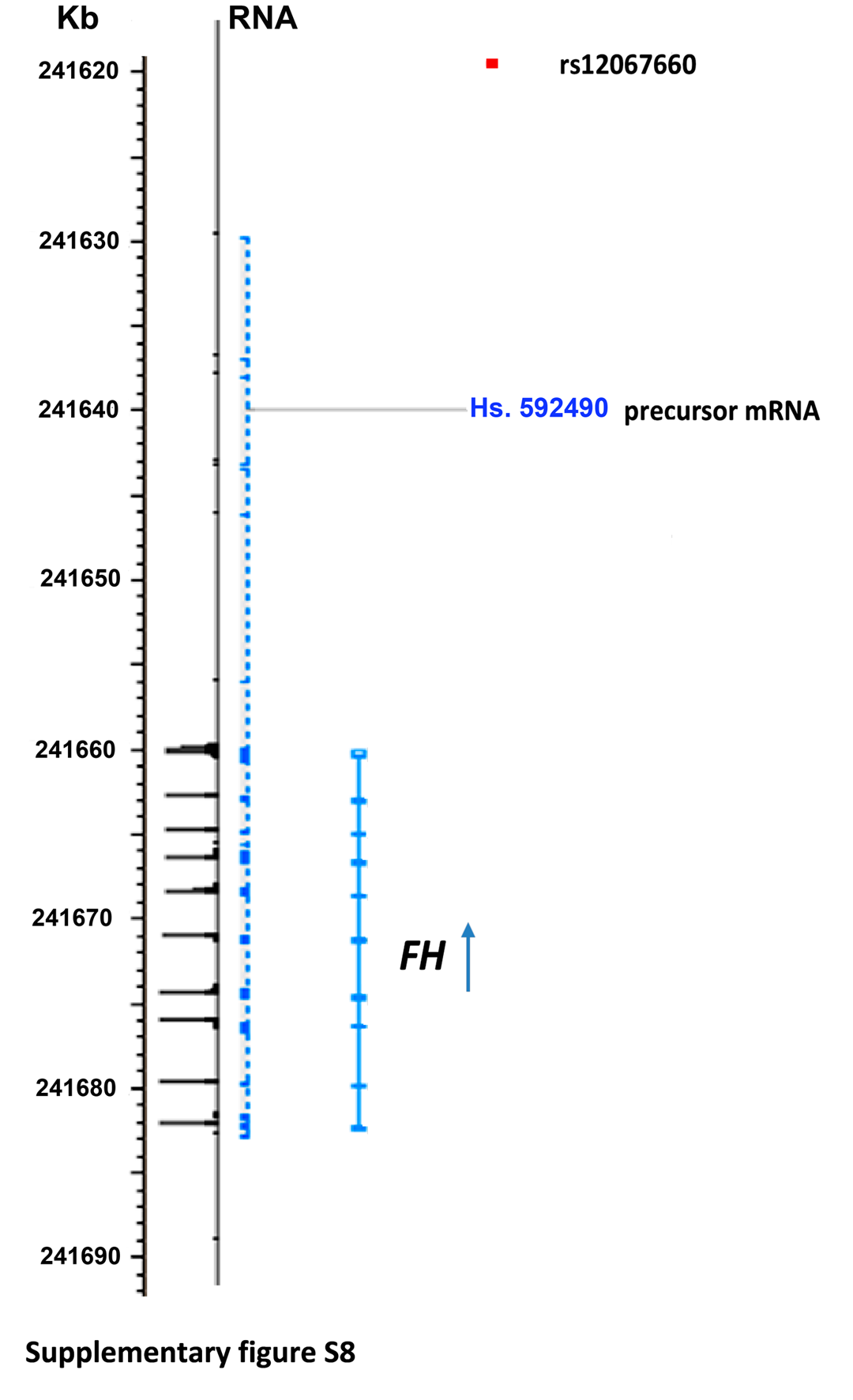

Supplement: Figure S8 — Genomic map of alternative splice variants of fumarate hydratase transcripts. The map shows the location of the precursor mRNA (Hs.592490) and the shorter splice variants of fumarate hydratase along with that of single nucleotide polymorphism rs12067660 found to be in significant association with the risk of uterine fibroids (p = 4.0×10^−4^; Fig. 1). (TIF) [file pone.0058399.s008.tif]
