# Supplementary material for: Multiple Hits for the Association of Uterine Fibroids on Human Chromosome 1q43
Source: PLoS One. 2013 Mar 14;8(3):e58399. doi: 10.1371/journal.pone.0058399 (PMC3604173; doi:10.1371/journal.pone.0058399)
Supplement: Table S1 — List of DNA variants associated* with the risk of uterine leiomyomas. (*) Only SNPs reaching significant levels of association (p≤0.01) in at least one of the race strata, African Americans (AA) or European Americans (EA), are reported. (ns) not significant at α = 0.05 in either EA or AA, or at α = 0.01 in both EA and AA. (nd) not determined. (no BMI) not adjusted for the effect of body mass index. (Upstream or downstream −2 Kb) SNP located within 2-kilobase distance from the transcription initiation site and the 3′ UTR, respectively. (§) Missense nucleotide substitution at codon 76 (Val76Ile). (¶) SNP rs10802996 was shown to be associated with cervical cancer [35]. (DOCX) [file pone.0058399.s009.docx]

**Table S1. List of DNA variants associated* with the risk of uterine leiomyomas**

| **SNP** | **position (bp)** | **inter-SNP distance** | **Gene** | **Variant function** | **p (AA)** | **p-(AA) no BMI** | **p (EA)** | **p (EA) no BMI** |
| --- | --- | --- | --- | --- | --- | --- | --- | --- |
| rs261802 | 241,065,464 |  | *RGS7* | intronic | 0.0062 | 0.0098 | - | - |
| rs16841104 | 241,106,858 | 41,394 | *RGS7* | intronic | - | 0.0066 | - | - |
| rs10926395 | 241,191,026 | 84,168 | *RGS7* | intronic | - | 0.0065 | - | - |
| rs10926396 | 241,191,811 | 785 | *RGS7* | intronic | 0.0018 | 0.0013 | - | - |
| rs12024588 | 241,280,303 | 88,492 | *RGS7* | intronic | 0.0093 | - | - | - |
| rs7550909 | 241,286,525 | 6,222 | *RGS7* | intronic | - | - | 0.0052 | - |
| rs9787056 | 241,287,348 | 823 | *RGS7* | intronic | 0.001 | 0.0014 | - | - |
| rs12126270 | 241,331,767 | 44,419 | *RGS7* | intronic | - | - | 0.0014 | - |
| rs6429249 | 241,335,069 | 3,302 | *RGS7* | intronic | - | - | 0.0017 | - |
| rs10802934 | 241,365,280 | 30,211 | *RGS7* | intronic | - | - | 0.0087 | - |
| rs4290050 | 241,399,513 | 34,233 | *RGS7* | intronic | - | - | - | 0.0083 |
| rs2341646 | 241,418,992 | 19,479 | *RGS7* | intronic | 0.0041 | 0.0018 | nd | - |
| rs4660080 | 241,542,793 | 123,801 | *RGS7-FH* | intergenic | 0.0061 | 0.0042 | - | 0.0459 |
| rs2341938 | 241,546,751 | 3,958 | *RGS7-FH* | intergenic | 0.0077 | 0.0060 | - | 0.0241 |
| rs6676068 | 241,548,330 | 1,579 | *RGS7-FH* | intergenic | 0.0059 | 0.0044 | - | 0.0300 |
| rs1557077 | 241,550,242 | 1,912 | *RGS7-FH* | intergenic | 0.0012 | 0.0030 | - | - |
| rs6429273 | 241,553,203 | 2,961 | *RGS7-FH* | intergenic | - | 0.0067 | - | - |
| rs12071612 | 241,553,587 | 384 | *RGS7-FH* | intergenic | 0.0013 | 0.0007 | - | - |
| rs10926477 | 241,555,823 | 2,236 | *RGS7-FH* | intergenic | 0.0038 | 0.0032 | - | - |
| rs10926480 | 241,565,538 | 9,715 | *RGS7-FH* | intergenic | 0.0073 | 0.0038 | 0.0474 | - |
| rs12093815 | 241,603,197 | 37,659 | *RGS7-FH* | intergenic | 0.0023 | 0.0032 | - | nd |
| rs12094946 | 241,603,419 | 222 | *RGS7-FH* | intergenic | 0.0023 | 0.0032 | - | nd |
| rs6691192 | 241,615,069 | 11,650 | *RGS7-FH* | intergenic | 0.0043 | 0.0058 | - | nd |
| rs12066083 | 241,619,685 | 4,616 | *RGS7-FH* | intergenic | 0.0029 | 0.0039 | - | nd |
| rs12067660 | 241,620,785 | 1,100 | *RGS7-FH* | intergenic | 0.0004 | 0.0008 | - | nd |
| rs2994979 | 241,633,731 | 12,946 | *precursor FH* | intronic | - | - | 0.005 | - |
| rs11586971 | 241,634,854 | 1,123 | *precursor FH* | intronic | - | - | 0.005 | - |
| rs12754512 | 241,634,905 | 51 | *precursor FH* | intronic | - | - | 0.005 | - |
| rs2994981 | 241,636,872 | 1,967 | *precursor FH* | intronic | - | - | 0.0045 | - |
| rs12566016 | 241,637,332 | 460 | *precursor FH* | intronic | 0.0527 | - | 0.0045 | - |
| rs10926494 | 241,637,536 | 204 | *precursor FH* | intronic | - | - | 0.0017 | - |
| rs2341939 | 241,643,459 | 5,923 | *precursor FH* | intronic | - | - | 0.0042 | - |
| rs2144 | 241,644,712 | 1,253 | *precursor FH* | intronic | - | - | 0.0014 | - |
| rs6429315 | 241,954,253 | 309,541 | *WDR64* | intronic | 0.0071 | - | nd | - |
| rs10802996 | 242,010,627 | 56,374 | *EXO1* | upstream-2Kb | - | - | - | 0.0094^¶^ |
| rs4149864 | 242,015,658 | 5,031 | *EXO1* | non-synonymous^§^ | - | 0.0089 | - | nd |
| rs4150028 | 242,053,998 | 38,340 | *EXO1* | down-tream-2Kb | - | 0.0093 | - | - |
| rs1776161 | 242,069,044 | 15,046 | *EXO1-MAP1LC3C* | intergenic | 0.0033 | 0.0074 | - | 0.0197 |
| rs6666851 | 242,081,840 | 12,796 | *EXO1-MAP1LC3C* | intergenic | - | 0.0099 | - | - |
| rs3845563 | 242,098,415 | 16,575 | *EXO1-MAP1LC3C* | intergenic | 0.0053 | 0.0048 | 0.0193 | - |
| rs10926736 | 242,603,322 | 504,907 | *PLD5* | intronic | - | - | - | 0.0030 |
| rs7531009 | 242,643,628 | 40,306 | *PLD5* | intronic | - | 0.0165 | - | 0.0012 |
| rs6429360 | 242,654,482 | 10,854 | *PLD5* | intronic | 0.0041 | 0.0025 | - | 0.0016 |
| rs10926756 | 242,662,040 | 7,558 | *PLD5* | intronic | - | - | - | 0.0093 |
| rs1039534 | 242,664,627 | 2,587 | *PLD5* | intronic | 0.0078 | 0.0062 | nd | - |
| rs10754775 | 242,871,018 | 206,391 | *PLD5 far upstream* | intergenic | - | - | - | 0.0038 |
| rs10926828 | 242,873,595 | 2,577 | *PLD5 far upstream* | intergenic | - | - | - | 0.0038 |
| rs6673294 | 242,874,466 | 871 | *PLD5 far upstream* | intergenic | - | - | - | 0.0095 |
| rs10803075 | 242,874,959 | 493 | *PLD5 far upstream* | intergenic | - | 0.0550 | - | 0.0075 |
| rs6679445 | 242,875,453 | 494 | *PLD5 far upstream* | intergenic | - | - | - | 0.0041 |
| rs7538080 | 242,886,970 | 11,517 | *PLD5 far upstream* | intergenic | - | - | 0.0056 | - |
