# Supplementary material for: Multiple Hits for the Association of Uterine Fibroids on Human Chromosome 1q43
Source: PLoS One. 2013 Mar 14;8(3):e58399. doi: 10.1371/journal.pone.0058399 (PMC3604173; doi:10.1371/journal.pone.0058399)
Supplement: Table S2 — List of DNA variants associated* with the growth of uterine leiomyomas. Data are from logistic models for uterine leiomyoma case-only design. (*) Only SNPs reaching significant levels of association (p≤0.01) in at least one of the race strata and meeting the assumption for proportional odds are reported. Of note, most of the SNPs that did not meet this assumption were not significant at α = 5%. (AA) African Americans; (EA) European Americans (EA). (ns) not significant at α = 5% in either EA or AA, or at α = 0.01 in both EA and AA. (nd) not determined. (no BMI) not adjusted for the effect of body mass index. (Upstream-2 Kb) SNP located within 2-kilobase distance from the transcription initiation site. (¶) synonymous nucleotide substitution at codon 23 (Cys23Cys). (DOCX) [file pone.0058399.s010.docx]

**Table S2. List of DNA variants associated* with the growth of uterine leiomyomas**

| **SNP** | **Position (bp)** | **inter-SNP distance** | **Gene** | **Variant function** | **p (AA)** | **p (AA) no BMI** | **p (EA)** | **p (EA) no BMI** |
| --- | --- | --- | --- | --- | --- | --- | --- | --- |
| rs7521765 | 240,988,389 |  | *RGS7* | intronic | - | - | - | 0.0047 |
| rs12410838 | 240,991,571 | 3,182 | *RGS7* | intronic | - | - | - | 0.0037 |
| rs12408427 | 241,001,045 | 9,474 | *RGS7* | intronic | 0.0065 | - | - | - |
| rs796786 | 241,003,220 | 2,175 | *RGS7* | intronic | 0.0021 | 0.0012 | - | - |
| rs261836 | 241,045,758 | 42,538 | *RGS7* | intronic | 0.0066 | - | nd | - |
| rs261833 | 241,046,905 | 1,147 | *RGS7* | intronic | - | 0.0034 | - | - |
| rs16841104 | 241,106,858 | 59,953 | *RGS7* | intronic | 0.0038 | 0.00008 | - | - |
| rs12068733 | 241,183,190 | 76,332 | *RGS7* | intronic | - | 0.0076 | - | - |
| rs9428858 | 241,237,942 | 54,752 | *RGS7* | intronic | - | - | - | 0.0082 |
| rs10926416 | 241,289,796 | 51,854 | *RGS7* | intronic | 0.0059 | - | - | - |
| rs6429246 | 241,334,960 | 45,164 | *RGS7* | intronic | 0.0031 | - | - | - |
| rs12025118 | 241,356,618 | 21,658 | *RGS7* | intronic | - | - | - | 0.0008 |
| rs2686226 | 241,376,512 | 19,894 | *RGS7* | intronic | - | - | - | 0.0031 |
| rs10926466 | 241,521,721 | 145,209 | *RGS7* | upstream-2Kb | 0.0018 | - | - | - |
| rs12038803 | 241,522,325 | 604 | *RGS7* | upstream-2Kb | 0.0065 | - | - | - |
| rs1341446 | 241,541,888 | 19,563 | *RGS7-FH* | intergenic | - | 0.0007 | - | - |
| rs4660080 | 241,542,793 | 905 | *RGS7-FH* | intergenic | - | 0.0042 | - | 0.0115 |
| rs2341938 | 241,546,751 | 3,958 | *RGS7-FH* | intergenic | - | 0.0038 | - | 0.0052 |
| rs6676068 | 241,548,330 | 1,579 | *RGS7-FH* | intergenic | - | 0.0033 | - | 0.0073 |
| rs1557078 | 241,550,350 | 2,020 | *RGS7-FH* | intergenic | - | - | - | 0.0057 |
| rs6429273 | 241,553,203 | 2,853 | *RGS7-FH* | intergenic | - | 0.0046 | - | 0.0224 |
| rs12071612 | 241,553,587 | 384 | *RGS7-FH* | intergenic | - | 0.0015 | - | - |
| rs10926480 | 241,565,538 | 11,951 | *RGS7-FH* | intergenic | - | 0.0052 | - | - |
| rs10926481 | 241,565,615 | 77 | *RGS7-FH* | intergenic | - | 0.0063 | - | - |
| rs2050927 | 241,574,624 | 9,009 | *RGS7-FH* | intergenic | - | - | 0.0095 | - |
| rs9428874 | 241,575,771 | 1,147 | *RGS7-FH* | intergenic | - | - | 0.0095 | - |
| rs1557080 | 241,576,926 | 1,155 | *RGS7-FH* | intergenic | - | - | 0.0089 | - |
| rs3014561 | 241,577,520 | 594 | *RGS7-FH* | intergenic | - | - | 0.0095 | - |
| rs1891129 | 241,586,687 | 9,167 | *RGS7-FH* | intergenic | - | - | - | 0.0069 |
| rs6657773 | 241,630,228 | 43,541 | *precursor FH* | intronic | 0.0093 | 0.00097 | - | - |
| rs3753220 | 241,777,806 | 147,578 | *OPN3* | intronic | - | - | 0.0061 | - |
| rs1537802 | 241,811,131 | 33,325 | *OPN3-WDR64* | intergenic | - | - | 0.0092 | - |
| rs10802976 | 241,814,114 | 2,983 | *WDR64* | upstream-2Kb | - | - | 0.0090 | - |
| rs4149857 | 242,013,826 | 199,712 | *EXO1* | synonymous | - | 0.0034^¶^ | - | nd |
| rs851781 | 242,034,868 | 21,042 | *EXO1* | intronic | - | 0.0088 | - | - |
| rs1776161 | 242,069,044 | 34,176 | *EXO1-MAP1LC3C* | intergenic | - | 0.0020 | - | 0.0508 |
| rs1635522 | 242,070,064 | 1,020 | *EXO1-MAP1LC3C* | intergenic | - | 0.0059 | - | 0.0591 |
| rs6700418 | 242,081,980 | 11,916 | *EXO1-MAP1LC3C* | intergenic | - | 0.0032 | - | - |
| rs3845563 | 242,098,415 | 16,435 | *EXO1-MAP1LC3C* |  | - | 0.0018 | - | - |
| rs4658576 | 242,102,508 | 4,093 | *EXO1-MAP1LC3C* |  | - | 0.0062 | - | - |
| rs1393299 | 242,329,791 | 227,283 | *PLD5* | intronic | - | - | 0.0084 | - |
| rs2036408 | 242,360,179 | 30,388 | *PLD5* | intronic | - | - | 0.0065 | 0.0092 |
| rs1021791 | 242,365,071 | 4,892 | *PLD5* | intronic | - | - | 0.0013 | 0.0022 |
| rs2653165 | 242,366,282 | 1,211 | *PLD5* | intronic | 0.0398 | - | 0.0049 | - |
| rs6682604 | 242,414,663 | 48,381 | *PLD5* | intronic | - | - | 0.0086 | - |
| rs316912 | 242,435,448 | 20,785 | *PLD5* | intronic | 0.0006 | - | nd | - |
| rs12731227 | 242,565,161 | 129,713 | *PLD5* | intronic | - | 0.0024 | - | - |
| rs2654881 | 242,600,263 | 35,102 | *PLD5* | intronic | - | - | 0.0016 | - |
| rs2654880 | 242,600,436 | 173 | *PLD5* | intronic | - | - | 0.0053 | - |
| rs2810025 | 242,600,468 | 32 | *PLD5* | intronic | - | - | 0.0042 | - |
| rs2654879 | 242,600,606 | 138 | *PLD5* | intronic | - | - | 0.0032 | - |
| rs2654884 | 242,601,406 | 800 | *PLD5* | intronic | - | - | 0.0043 | - |
| rs10926736 | 242,603,322 | 1,916 | *PLD5* | intronic | - | - | - | 0.0008 |
| rs1039534 | 242,664,627 | 61,305 | *PLD5* | intronic | - | 0.0004 | - | - |
| rs10754775 | 242,871,018 | 206,391 | *PLD5 far upstream* | intergenic | - | - | - | 0.0011 |
| rs10926828 | 242,873,595 | 2,577 | *PLD5 far upstream* | intergenic | - | - | - | 0.0013 |
| rs6679445 | 242,875,453 | 1,858 | *PLD5 far upstream* | intergenic | - | - | 0.0076 | 0.0014 |
| rs1996626 | 242,876,648 | 1,195 | *PLD5 far upstream* | intergenic | - | - | 0.0045 | - |
| rs10803076 | 242,881,445 | 4,797 | *PLD5 far upstream* | intergenic | - | - | 0.0079 | - |
| rs2027047 | 242,911,065 | 29,620 | *PLD5 far upstream* | intergenic | 0.0064 | - | - | - |
